# Effects of accelerated rehabilitation after ACL reconstruction on clinical and functional outcomes: A systematic review and meta‐analysis evaluating clinical meaningfulness

**DOI:** 10.1002/jeo2.70878

**Published:** 2026-08-06

**Authors:** Mostafa Jalili Bafrouei, Hooman Minoonejad, Rahman Sheikhhoseini, Seyed Hamed Mousavi

**Affiliations:** ^1^ Department of Sport Injuries and Biomechanics, Faculty of Sport Sciences and Health University of Tehran Tehran Iran; ^2^ Department of Corrective Exercise and Sport Injury, Faculty of Physical Education and Sport Sciences Allameh Tabataba'i University Tehran Iran

**Keywords:** accelerated rehabilitation, ACLR, clinical meaningful, knee stability

## Abstract

**Purpose:**

Pain, joint laxity, deficits in functional performance and proprioception are common after anterior cruciate ligament reconstruction (ACLR). The role of accelerated rehabilitation (AR) in addressing these outcomes remains uncertain. This systematic review and meta‐analysis aimed to evaluate the effects of AR on laxity, functional performance, proprioception and pain in individuals post‐ACLR.

**Method:**

Four databases (PubMed, Web of Science, Scopus, Embase) were searched until March 2026. Eligible interventional studies comparing AR with standard exercise in individuals post‐ACLR were included. Data extraction followed Preferred Reporting Items for Systematic Reviews and Meta‐Analyses (PRISMA) 2020 guidelines. Risk of bias was assessed using the PEDro and Newcastle–Ottawa scales. Knee pain and laxity were defined as the primary outcome, with functional performance and proprioception evaluated as secondary outcomes. Random‐effects models calculated mean differences (MDs) or standardized mean differences (SMDs) with 95% confidence intervals (CIs).

**Results:**

Ten studies (*n* = 2180 participants) were included. AR resulted in statistically significant improvements in knee laxity (MD: −0.50; 95% CI: −0.65 to −0.36; *p* < 0.001), proprioception (SMD: 0.20; 95% CI: 0.10–0.30; *p* < 0.001) and performance outcomes, such as single‐leg hop (SMD: 0.85; 95% CI: 0.43–1.27; *p* < 0.001), International Knee Documentation Committee (MD: 2.39; 95% CI: 0.74–4.03; *p* = 0.004) and Lysholm score (MD: 1.54; 95% CI: 0.01–3.07; *p* = 0.04). However, these changes did not reach established thresholds for clinical meaningfulness, and the knee pain outcome (SMD: −0.14, 95% CI: −0.54 to 0.24, *p* = 0.45) showed no significant changes.

**Conclusion:**

AR was associated with statistically detectable changes in knee laxity, functional outcomes and knee proprioception; however, these effects had limited clinical relevance and did not extend to pain reduction. Therefore, the clinical value of AR in optimizing post‐ACLR recovery remains uncertain.

**Level of Evidence:**

Level I.

**Abbreviations**
ACL: anterior cruciate ligament
ACLR: anterior cruciate ligament reconstruction
ADL: Activities of Daily Living
AE: acceleration exercises
CI: confidence interval
IKDC: International Knee Documentation Committee
LSI: Limb Symmetry Index
QOL: quality of life
VAS: Visual Analogue Scale

## INTRODUCTION

Anterior cruciate ligament (ACL) injuries frequently require surgical reconstruction to restore knee stability and function [10, 48, 62]. Although ACL reconstruction (ACLR) effectively restores anatomical knee stability [7], persistent deficits in functional performance and proprioception remain common following surgery [11, 63]. These residual impairments may adversely affect recovery and long‐term function despite advances in surgical and rehabilitation approaches.

Following ACLR, rehabilitation is essential for regaining muscular strength, proprioception and functional performance [15]. Persistent neuromuscular deficits [37], muscle imbalances [9, 54] and inadequate rehabilitation progression may limit recovery of pre‐injury functional capacity [57]. These limitations can compromise joint stability and movement quality despite successful reconstruction [3].

Poor functional recovery after ACLR may be associated with inadequate exercise progression and persistent neuromuscular deficits during rehabilitation [15, 21, 33]. Insufficient rehabilitation progression may limit improvements in neuromuscular function and strength, contributing to functional asymmetries [39, 44]. Furthermore, persistent deficits in knee proprioception, dynamic balance, strength and pain perception may hinder the restoration of pre‐injury physical function [24, 56, 64].

Given their potential effects on physical function and neuromuscular adaptations, accelerated rehabilitation (AR) may represent a relevant component of post‐ACLR rehabilitation [31]. AR may facilitate earlier progression of rehabilitation activities and potentially improve neuromuscular function and physical performance after ACLR [51]. AR has been proposed as a valuable component of ACLR rehabilitation due to its potential effects on proprioception, neuromuscular function and physical performance [30, 53]. These mechanisms may contribute to improved knee‐related outcomes during recovery.

The effects of AR following ACLR remain unclear. Previous studies have reported inconsistent findings regarding functional outcomes, proprioception and knee‐related recovery after ACLR [1, 5, 6]. Moreover, quantitative synthesis of the available evidence remains limited [30]. Therefore, this systematic review and meta‐analysis aimed to evaluate the effects of AR, primarily characterized by earlier progression of rehabilitation activities following ACLR, compared with standard rehabilitation on pain, knee laxity, functional performance and proprioception outcomes.

## METHOD

### Protocol and registration

This systematic review and meta‐analysis was conducted and reported according to the Preferred Reporting Items for Systematic Reviews and Meta‐Analyses (PRISMA) 2020 statement guidelines [31]. The protocol was prospectively registered with PROSPERO (CRD42025634130) before data extraction and analysis.

### Search strategy

Relevant studies were identified through a comprehensive search of four electronic databases: PubMed, Web of Science, Scopus and Embase, conducted on 15 March 2026. The search strategy was based on broad terms and related synonyms organized: #1 ‘anterior cruciate ligament’ OR ACL OR ‘anterior cruciate ligament reconstruction’ OR ‘ACL reconstruction’ OR ACLR OR ACL‐R OR ACL‐surgery, #2 ‘accelerat* rehabilit*’ OR ‘accelerat* exercise’ OR ‘accelerat* train*’ OR ‘accelerat* protocol’ or ‘accelerat* intervent*’ OR accelerat*, #3 function* OR pain OR activity OR laxity OR propriocept* OR brace OR biomechanic*, #4 (1 AND 2 AND 3). Additionally, Google Scholar and reference lists from previous systematic reviews on AR in ACLR populations were manually searched to ensure the inclusion of all relevant studies.

### Eligibility criteria

We included peer‐reviewed interventional studies (randomized controlled trials [RCTs] and non‐randomized studies) assessing AR intervention according to the following criteria (PICOs):

Population: Participants undergoing ACLR, aged 12–53 years, regardless of sex or graft type. Eligible studies included patients in the early postoperative rehabilitation phase, with AR protocols initiated immediately after ACLR. Studies including patients with previous ligament surgery, revision ACLR, multi‐ligament injuries or concomitant/contralateral ligament injuries or procedures were excluded.

Intervention: AR was defined as any rehabilitation protocol following ACLR that advanced exercise progression, weight‐bearing activities, strengthening exercises, neuromuscular training, functional tasks or return‐to‐activity milestones earlier than conventional rehabilitation protocols. The definition was not restricted to a specific exercise modality but was based on the earlier initiation or progression of rehabilitation components relative to standard care. Eligible interventions aimed to improve knee pain, biomechanical outcomes, functional performance and proprioception following ACLR.

Comparators: Any control intervention (e.g., traditional exercise, rehabilitation, standard exercise or physiotherapy standard).

Outcomes: The primary outcomes measured were pain intensity and knee laxity, assessed using the Visual Analogue Scale (VAS), Knee Injury and Osteoarthritis Outcome Score (KOOS)‐pain and the KT‐1000, respectively. The KT‐1000 arthrometer was selected because it provides an objective measure of anterior tibial translation and is widely used to assess anterior knee joint laxity following ACLR. Secondary outcomes included proprioception, which was assessed using the joint position sense test, and functional performance using a validated questionnaire such as the International Knee Documentation Committee (IKDC), Tegner scale, KOOS‐Activities of Daily Living (ADL), Lysholm score and single‐leg hop test.

Study design: Any interventional studies that have a control group (RCTs and non‐randomized studies).

Study design: Interventional studies with comparison groups (RCTs and non‐randomized).

Language and publication status: Peer‐reviewed articles published in English.

Studies involving cross‐sectional, case‐control, cohort, animal models, conference abstracts, editorials, reviews or case studies were excluded.

### Study selection

All records identified through the search strategy were imported into the EndNote software (Version 21.3), and duplicates were removed. Titles and abstracts were independently screened by M. J. B. and H. M. for relevance, followed by full‐text assessment of potentially eligible articles against predefined inclusion criteria. Discrepancies between reviewers were resolved through consensus or consultation with a third reviewer (R. S. H.).

### Methodological quality assessment

The methodological quality of the included studies was assessed by M. J. B. using the PEDro checklist, which comprises 11 items [17] for RCTs. The PEDro Score has demonstrated inter‐rater reliability ranging from ‘fair’ to ‘excellent’ (intraclass correlation [ICC] = 0.53 to 0.91) and ‘excellent’ inter‐rater reliability (ICC = 0.80 to 0.89) for clinical trials related to physiotherapy interventions [49]. Scores are categorized as follows: <4 is considered ‘poor,’ 4–5 is ‘fair,’ 6–8 is ‘good’ and 9–10 is ‘excellent’ [49]. Disagreements were resolved via consensus‐based discussion when necessary. The Newcastle–Ottawa scale (NOS) is a tool used for assessing the quality of non‐randomized studies [59]. Disagreements between reviewers were resolved through discussion and consensus.

### Data extraction

M. J. B. and H. M. extracted all data independently from the included studies. Information on the number of sessions, study design, variables, interventions, number of participants and their characteristics (age, sex, height, mass), AR protocols and the measurement instruments used to assess outcomes was systematically extracted from the included studies. To ensure data accuracy and completeness, R. S. H. and S. H. M. independently verified all extracted data. Any discrepancies identified during the extraction or verification process were resolved through discussion and consensus among the authors.

### Statistical analysis

Mean differences or standard mean differences and 95% confidence intervals (CIs) were calculated using a random‐effects model in CMA version 7.4. Meta‐analysis was performed when at least three studies reported comparable outcomes using similar methodologies. Statistical heterogeneity was assessed using the *I* ^2^ statistic, with values of 25%, 50% and 75% representing low, moderate and high heterogeneity, respectively. To assess the robustness of the meta‐analytic findings, a sensitivity analysis was performed. A one‐study‐removed approach was applied, in which each study was sequentially excluded, and the pooled effect size and 95% CI were recalculated using a random‐effects model. A random‐effects model was chosen due to expected clinical heterogeneity (differences in participant characteristics, interventions and follow‐up periods) and methodological heterogeneity (variations in study design, outcome measures and assessment tools). The results were examined to determine whether the overall conclusions were influenced by the inclusion of any single study.

The results were interpreted based on the levels of evidence established by Tulder et al., as modified by Bafrouei et al. [9], which provides guidelines for conducting and reporting systematic reviews in this research domain (Table S1).

### Assessment of publication bias

Publication bias was assessed using visual inspection of funnel plots, Begg's test, Egger's regression test and trim‐and‐fill analysis when applicable. For meta‐analyses including ≥10 studies, Begg's test, Egger's regression test and trim‐and‐fill methods were performed [60]. Due to the limited number of included studies, the results of publication bias assessments were interpreted with caution because of low statistical power.

### Missing data

Authors were not contacted to obtain missing or unpublished data. Therefore, only data available in the published reports were included in the analyses.

### Narrative synthesis

Outcomes with fewer than three studies providing comparable data were synthesized narratively. Narrative synthesis followed a structured approach describing study characteristics, participant demographics, measurement methods and reported effects with direction and magnitude where available. Where possible, we calculated effect sizes for individual studies to facilitate interpretation.

### GRADE (Grading of Recommendations, Assessment, Development and Evaluations) assessment

The GRADE framework was employed to assess the quality of evidence for the outcomes reported in this review [35]. The GRADE approach evaluates evidence based on five domains: risk of bias, inconsistency, indirectness, imprecision and publication bias. Additionally, it considers three factors that may increase the quality of evidence: large effect size, dose‐response gradient and plausible confounding [32]. Two authors conducted the assessment independently, with disagreements resolved through discussion or consultation with a third reviewer.

## RESULTS

### Study selection

**Figure 1:**
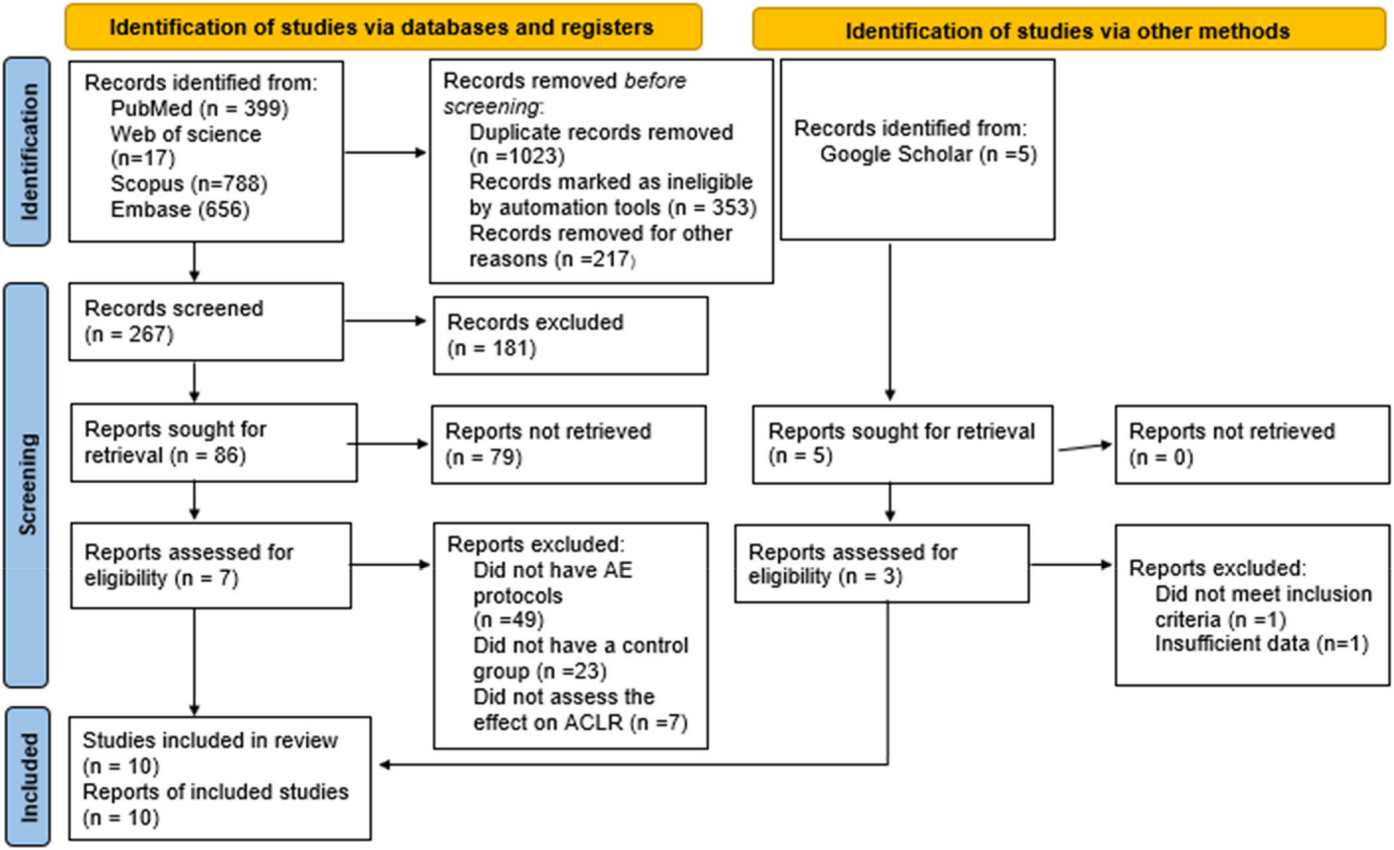
Flowchart of study selection process. ACLR, anterior cruciate ligament reconstruction; AE, acceleration exercises.

The initial literature search identified 1,860 records (PubMed: 399; Scopus: 788; Embase: 656; Web of Science: 17). After duplicate removal (*n* = 1023), records marked as ineligible by automation tools (*n* = 353) and records removed for other reasons (*n* = 217), a total of 267 records proceeded to title and abstract screening, with 10 studies meeting the inclusion criteria. Figure 1 shows the flow diagram of the selection process and the number of excluded studies at each stage. The primary exclusion criteria comprised: absence of an established AR protocol (*n* = 49), lack of a control group (*n* = 23) and failure to assess ACLR‐related (*n* = 7).

### Study characteristics

Table 1 shows the characteristics of the included studies. Publication years ranged from 1992 to 2024 (median: 2007), with 90% (9/10) of the studies published after 2007. The 10 eligible studies included five RCTs and five non‐randomized. Moreover, these studies enroled 2180 participants. The inconsistent reporting of graft type across studies limits the ability to explore graft‐specific acceleration outcomes following ACLR.

**Table 1:** Study characteristics for included studies.

Authors	Study design	Duration	Participants	Time post‐ACLR and type of graft	Intervention	Outcome	Measurement instruments	AR protocols	Funding and conflict of interest	Sample size and sex	Age (year)	Weight (kg)	Height (cm)	BMI (kg/m^2^)
Elabd et al. 2024 [29]	RCT	22 Weeks (5 sessions per week)	ACLR	Early phase, HG	EG: AR CG: standard rehabilitation	Pain, function	VAS, single‐hop test battery, KOOS and knee effusion scale	Phase‐based rehabilitation programme (four phases) initiated immediately after ACLR, emphasizing early full weight‐bearing, immediate ROM restoration, progressive quadriceps and hamstring strengthening, proprioceptive and neuromuscular training, balance/stability exercises and sport‐specific activities. Training was performed with progressive load increases (2%–10%) based on patient tolerance and pain monitoring (VAS ≤ 5).	King Saud University, Riyadh, Saudi Arabia (RSP2024R382)	EG: 50 males, CG: 50 males	EG: 21.92 ± 1.74, CG: 22.09 ± 1.83	EG: 71.62 ± 4.68, CG: 71.13 ± 4.63	EG: 1.72 ± 0.04, CG: 1.72 ± 0.04	EG: 24.07 ± 0.90, CG: 24.13 ± 0.89
Deichsel et al. 2023 [26]	Non‐randomized	12 Weeks (NR sessions per week)	ACLR	Early phase, HG	EG: AR CG: standard rehabilitation	Pain, function	VAS, IKDC, Tegner score and Lysholm score	Early brace‐free protocol initiated 5 days after surgery, including immediate ROM exercises, early progression to full weight‐bearing and closed‐chain strengthening from the early postoperative phase. Proprioceptive/dynamic stability training started at 4 weeks, running at 6 weeks and return to pivoting sports after ≥8 months based on RTS testing.	The University of Münster, Germany	EG: 8 females, 25 males, CG: 18 females, 14 males	EG: 29.3 ± 11.7, CG: 26.6 ± 11.7	NR	NR	EG: 24.1 ± 2.6, CG: 23.6 ± 3.9
Ebert et al. 2022 [28]	RCT	24 Months (2–3 sessions per week)	ACLR	Early phase, HG	EG: AR CG: standard rehabilitation	Laxity, knee extensor and flexor strength and function	KT‐1000, isokinetic and single‐leg hop test	Criterion‐based progressive rehabilitation with earlier introduction of strengthening, proprioceptive, plyometric, running and agility exercises compared with standard rehabilitation. The programme combined supervised and home‐based training and progressed according to quadriceps control, pain/effusion, ROM, movement competency and functional performance milestones. RTS activities were initiated earlier, with RTS recommended after ≥9 months and achievement of objective functional criteria.	Smith and Nephew to assist this research	EG: 22 males, CG: 21 males	EG: 24.9 ± 7.1, CG: 25.7 ± 7.9	NR	NR	EG: 25.3 ± 2.4, CG: 25.1 ± 3.3
Cristiani et al. 2020 [23]	RCT	24 Weeks (2–3 sessions per week)	ACLR	Early phase, BPTBG and HG	EG: AR CG: standard rehabilitation	Quadriceps and hamstring strength at 90°/s and the SLH test performance	Isokinetic, LSI	Early progression of rehabilitation milestones, including achievement of full ROM and weight‐bearing within 1–3 weeks, earlier initiation of open‐kinetic‐chain strengthening, running, plyometric exercises, perturbation training and sport‐specific drills, compared with the standard protocol. Supervised rehabilitation was performed, and predefined functional milestones guided progression.	Karolinska Institute	EG: 54 males and 26 females, CG: 61 males and 19 females	EG: 28.5 ± 5.5; CG: 29.3 ± 6.4	NR	NR	NR
Gupta et al. 2017 [34]	Non‐randomized	EG: 19 weeks (NR sessions per week), CG: 24 weeks (NR sessions per week)	ACLR	Early phase, HG	EG: AR CG: standard rehabilitation	Function, laxity	SLH test, IKDC score, KOOS score, Tegner scale, Lachman test and Pivot shift test	A 19‐week ARs compared with a 24‐week standard protocol. Both protocols used similar rehabilitation components, but exercise progression, ROM progression, weight‐bearing, brace use and functional activities were introduced at different time points in the accelerated group.	NA	EG: 20 males, CG: 18 males, 2 females	EG: 26.45 ± 4.69, CG: 28.90 ± 6.30	NR	NR	NR
Lee et al. 2016 [46]	Non‐randomized	12 Weeks (5 sessions per week)	ACLR	Early phase, NR	EG: AR CG: standard rehabilitation	Knee muscle strength, function, balance	Isokinetic, thigh circumference, Lysholm score and active balance agility scale	A four‐phase rehabilitation programme was initiated 2 days after ACLR. Phase 1 focused on oedema/inflammation control and muscle activation (1–2 weeks); Phase 2 on ROM restoration and early functional exercises (2–4 weeks); Phase 3 on progressive strengthening (4–8 weeks); and Phase 4 on balance and agility improvement (8–12 weeks). Exercise intensity and progression were adjusted according to pain level and patient compliance.	NA	EG: 8 males, CG: 8 males	EG: 18.00 ± 1.71, CG: 18.14 ± 1.21	EG: 74.85 ± 3.27, CG: 73.00 ± 2.64	EG: 173.85 ± 5.46, CG: 174.28 ± 5.03	NR
An et al. 2016 [6]	Non‐randomized	24 Weeks (2–3 sessions per week)	ACLR	Early phase, NR	EG: AR CG: standard rehabilitation	Proprioception	Isokinetic dynamometer	Initiated immediately after ACLR. The programme emphasized progressive restoration of knee function, proprioception and dynamic balance.	NA	EG: 10 males, 8 females, CG: 10 males, 8 females	EG: 29.2 ± 7.2, CG: 24.8 ± 5.8	EG:72.3 ± 9.6, CG: 72.3 ± 9.6	EG: 175.3 ± 5.7, CG: 175.3 ± 5.7	EG: 23.9 ± 2.6, CG: 22.1 ± 2.3
Christensen et al. 2013 [20]	RCT	24 Weeks (NR sessions per week)	ACLR	Early phase, HG	EG: AR CG: standard rehabilitation	Antero‐posterior knee laxity, range of motion and peak isometric force, function	KT1000 arthrometer (MEDmetric Corp), dual‐arm goniometer, Isokinetic, IKDC	Immediate postoperative protocol with unrestricted knee ROM, early hyperextension restoration, CPM use (0°−50°) and weight‐bearing as tolerated from Day 1 without bracing. Rehabilitation progressed through four time‐based phases: Phase I (0–4 weeks) focused on ROM recovery and muscle activation; Phase II (4–8 weeks) on strengthening, neuromuscular control and functional activities; Phase III (8–12 weeks) on advanced strengthening, neuromuscular training and running; and Phase IV (12–24 weeks) on sport‐specific neuromuscular training, plyometrics, sprinting and cutting drills.	NA	EG: 8 males, 7 females, CG: 14 males, 1 female	EG: 30.1 ± 10.5, CG: 33.1 ± 10.9	EG: 77.02 ± 10.5, CG: 80.65 ± 13.0	EG: 174.2 ± 7.5, CG: 176.0 ± 6.6	EG: 25.45 ± 3.82, CG: 25.94 ± 3.32
Beynnon et al. 2011 [13]	RCT	EG: 19 weeks (3 sessions per week), CG: 32 weeks (3 sessions per week)	ACLR	Early phase, BPTBG	EG: AR CG: standard rehabilitation	Pain, function, knee strength, proprioception	KOOS‐pain, IKDC, single‐leg hop test, Tegner score, isokinetic, goniometer	A biomechanically driven (earlier graft loading) rehabilitation programme delivered over 19 weeks (vs. 32 weeks in the control group), with earlier progression of weight‐bearing, discontinuation of crutch use, quadriceps‐dominant exercises and return‐to‐activity tasks to increase graft loading during rehabilitation.	The National Institutes of Health (Grants R01 AR051477‐01 T32 AR07568)	EG: 13 males, 6 females, CG: 9 males, 8 females	EG: 29.7 ± 10.1, CG: 30.2 ± 9.9	EG: 75.3, CG: 71.8	NR	NR
De Carlo et al. 1992 [25]	Non‐randomized	24 weeks	ACLR	Early phase, BPTBG	EG: AR CG: standard rehabilitation	Laxity, hamstring and quadriceps strength, proprioception	KT‐1000, Isokinetic, goniometer	A phase‐based accelerated ACLR rehabilitation protocol initiated immediately after surgery, emphasizing early ROM restoration, terminal knee extension, early weight‐bearing and closed kinetic chain strengthening. CPM and Cryo/Cuff were started immediately postoperatively. Rehabilitation progressed from ROM and muscle activation (first 1–3 weeks) to strengthening, proprioception, agility, running and sport‐specific activities. Progression was guided by ROM achievement, quadriceps strength (isokinetic testing) and functional performance criteria.	NA	EG: 1052, CG: 600 (males and females)	12 to 53	NR	NR	NR

Abbreviations: ACLR, anterior cruciate ligament reconstruction; AR, accelerated rehabilitation; BPTBG, bone–patellar tendon–bone graft; CG, control group; CPM, continuous passive motion; EG, experimental group; HG, hamstring graft; IKDC, International Knee Documentation Committee; KOOS, Knee Injury and Osteoarthritis Outcome Score; LSI, Limb Symmetry Index; NA, not available; NR, not reported; RCT, randomized controlled trial; ROM, range of motion; RTS, return‐to‐sport; SLH, single‐leg hop; VAS, Visual Analogue Scale.

### Methodological quality assessment

The PEDro scores ranged from 5/10 to 8/10, with the highest scores reported in Beynnon et al., Cristiani et al. and Elabd et al., and the lowest score observed in Christensen et al. NOS ratings ranged from 5/9 to 8/9; the highest scores were reported in An et al. and Lee et al., while De Carlo et al. and Deichsel et al. generally received the lowest ratings (5/9) (Table S2). Methodological quality and risk of bias were independently assessed using the Risk of Bias (RoB) 2 tool [16] for randomized trials and the Risk Of Bias In Non‐randomized Studies—of Interventions (ROBINS‐I) [17] for non‐randomized studies, and discrepancies were resolved through consensus (Table S3). GRADE assessment showed low certainty of evidence for the IKDC, Lysholm score, KOOS‐ADL, knee laxity and proprioception outcomes. The certainty of evidence was downgraded primarily due to concerns regarding the risk of bias associated with the inclusion of studies with different methodological designs (randomized and non‐randomized studies). Very low certainty of evidence was observed for the single‐leg hop test, pain and Tegner score outcomes due to additional concerns related to inconsistency for the single‐leg hop test (*I* ^2 ^= 50%) and imprecision resulting from non‐significant findings and limited certainty of effect estimates for pain and Tegner score outcomes. Detailed GRADE assessments for all outcomes are presented in Table S4.

### Sensitivity analysis

Sensitivity analyses were performed using a leave‐one‐out approach to assess the robustness of the pooled findings. For pain, exclusion of Deichsel et al. [26] changed the result from non‐significant to significant (*p* = 0.04). For knee laxity, removal of Shelbourne et al. [58] resulted in a non‐significant pooled effect (*p* = 1.00), indicating that this study had a substantial influence on the overall estimate. For IKDC scores, exclusion of Gupta et al. [34] altered the statistical significance of the pooled effect, resulting in a non‐significant finding (*p* = 0.12).

For KOOS‐ADL, sensitivity analyses identified two influential studies. Exclusion of Elabd et al. [29] resulted in loss of statistical significance (*p* = 0.30), while the exclusion of Gupta et al. [34] also attenuated the pooled effect, resulting in a borderline non‐significant finding (*p* = 0.06). For Lysholm scores, removal of Deichsel et al. [26] (*p* = 0.12) and Ebert et al. [28] (*p* = 0.19) influenced the stability of the pooled findings. For the Tegner activity scale, exclusion of Deichsel et al. [26] resulted in a statistically significant effect (*p* = 0.014), whereas the overall meta‐analysis remained non‐significant (*p* = 0.054).

For knee proprioception, removal of De Carlo et al. (*p* = 0.33) [25] and An et al. [6] (*p* = 0.10) resulted in loss of statistical significance, suggesting that the pooled estimate was sensitive to these studies. In contrast, the pooled effect for the single‐leg hop test remained unchanged after excluding individual studies, indicating a robust and stable finding (Table S5).

### Publication bias

Publication bias was assessed using Egger's and Begg's tests. No evidence of publication bias was observed for pain, knee laxity, IKDC, KOOS‐ADL, Lysholm, Tegner, single‐leg hop test and knee proprioception outcomes. Due to the limited number of studies included in each meta‐analysis, publication bias findings should be interpreted cautiously.

### Outcome measured

Of the 10 studies [6, 13, 20, 23, 26, 28, 29, 34, 46, 58], seven measured performance [13, 20, 26, 28, 29, 34, 46], four measured proprioception [6, 13, 46, 58], three targeted biomechanics [20, 28, 58] and three assessed pain [13, 26, 29].

#### Effects of AR on knee pain

**Figure 2:**
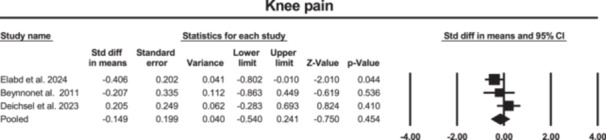
Forest plot regarding the comparison of the accelerated rehabilitation group (left) and the standard rehabilitation group (right) in knee pain. CI, confidence interval; Std, standard.

Three studies investigated the effect of the AR on knee pain, using the KOOS‐pain subscale and the VAS [13, 26, 29]. Pooled analysis revealed no statistically or clinically meaningful reduction in pain (standardized mean difference [SMD]: −0.14, 95% CI: −0.54 to 0.24, *p* = 0.45) with moderate heterogeneity (*I* ^2 ^= 45%, *p* = 0.16) (Figure 2).

#### Effects of AR on knee laxity

**Figure 3:**
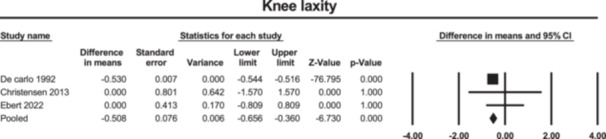
Forest plot regarding the comparison of the accelerated rehabilitation group (left) and the standard rehabilitation group (right) in knee laxity. CI, confidence interval.

Three studies investigated the effect of AR on knee anterior‐posterior laxity using the KT‐1000 score [20, 28, 58]. The results of the meta‐analysis suggested a decrease in knee anterior‐posterior laxity (mean difference [MD]: −0.50; 95% CI: −0.65 to −0.36; *p* < 0.001) with low heterogeneity (*I* ^2 ^= 4%, *p* = 0.35) (Figure 3). While the meta‐analysis showed a statistically significant change in KT‐1000 laxity (MD: −0.50 mm), this change is well below the established minimal clinically important difference (MCID) of 1.5 mm, indicating that the improvement is unlikely to be clinically meaningful for patients [18].

#### Effects of AR on performance

Seven studies investigated the effect of AR on performance [13, 20, 26, 28, 34, 46], according to IKDC [13, 20, 26, 28, 34], KOOS‐ADL [13, 29, 34], single‐leg hop test [13, 28, 29, 34, Tegner score [13, 26, 28, 34 and Lysholm score [26, 28, 46].

##### IKDC

**Figure 4:**
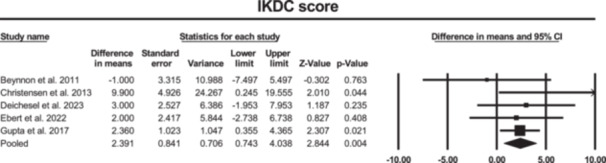
Forest plot regarding the comparison of the accelerated rehabilitation group (left) and the standard rehabilitation group (right) in the IKDC score. CI, confidence interval; IKDC, International Knee Documentation Committee.

The results of the meta‐analysis suggested an increase in IKDC scores (MD: 2.39; 95% CI: 0.74–4.03; *p* = 0.004) with no evidence of heterogeneity (*I* ^2 ^= 0%, *p* = 0.48) (Figure 4). Although statistically significant, the pooled MD of 2.39 points is considerably lower than the previously reported MCID for the IKDC score (6.3–11.5 points) [12, 18, 52].

##### KOOS‐ADL

**Figure 5:**
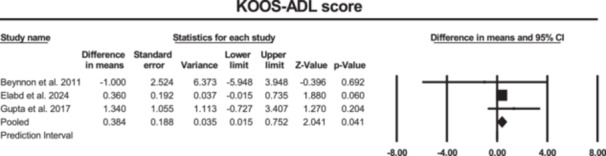
Forest plot regarding the comparison of the accelerated rehabilitation group (left) and the standard rehabilitation group (right) in the KOOS‐ADL score. CI, confidence interval; KOOS‐ADL, Knee Injury and Osteoarthritis Outcome Score—Activities of Daily Living.

The results of the meta‐analysis showed an increase in the KOOS‐ADL (MD: 0.38; 95% CI: 0.01–0.75; *p* = 0.04) with no heterogeneity (*I* ^2 ^= 0%, *p* = 0.56) (Figure 5). Despite statistical significance, the pooled MD of 0.38 points is substantially lower than the established MCID for KOOS‐ADL (8–13.3 points) [12], indicating no clinically meaningful benefit.

##### The Lysholm score

**Figure 6:**
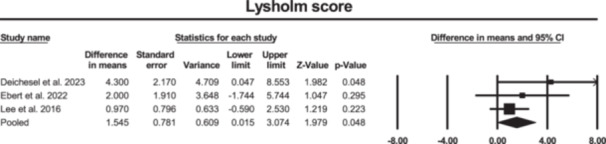
Forest plot regarding the comparison of the accelerated rehabilitation group (left) and the standard rehabilitation group (right) in the Lysholm score. CI, confidence interval.

The meta‐analysis suggested an increase in Lysholm scores (MD: 1.54; 95% CI: 0.01–3.07; *p* = 0.04) with low heterogeneity (*I* ^2 ^= 8%, *p* = 0.33) (Figure 6). Heterogeneity was minimal, indicating that variability across studies was negligible. Although the result was statistically significant, the pooled MD of 1.54 points is considerably lower than the reported MCID for the Lysholm score post‐ACLR (8.9–10.1 points) [12, 18, 52].

##### Single‐leg hop

**Figure 7:**
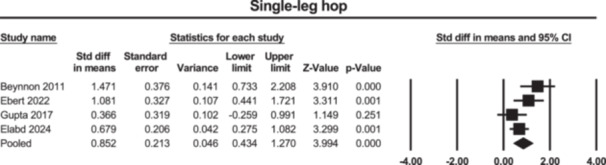
Forest plot regarding the comparison of the accelerated rehabilitation group (left) and the standard rehabilitation group (right) in the single‐leg hop test. CI, confidence interval; Std, standard.

Meta‐analysis showed an increase in the single‐leg hop (SMD: 0.85; 95% CI: 0.43–1.27; *p* < 0.001) with moderate heterogeneity (*I* ^2 ^= 50%, *p* = 0.10) (Figure 7). Although the result statistically showed a significant difference in the single‐leg hop test, a specific MCID threshold for this outcome has not yet been established [16].

##### Tegner score

**Figure 8:**
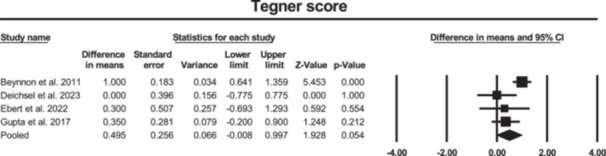
Forest plot regarding the comparison of the accelerated rehabilitation group (left) and the standard rehabilitation group (right) in the Tegner score. CI, confidence interval.

The meta‐analysis showed no significant change in Tegner score (MD: 0.49; 95% CI: −0.00 to 0.99; *p* = 0.054) with moderate heterogeneity (*I* ^2 ^= 62%, *p* = 0.04) (Figure 8).

#### Effects of AR on knee proprioception

**Figure 9:**
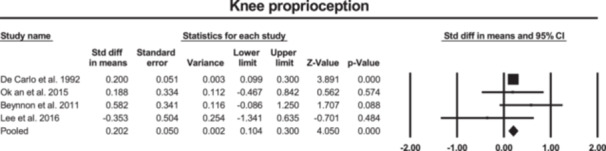
Forest plot regarding the comparison of the accelerated rehabilitation group (left) and the standard rehabilitation group (right) in knee proprioception. CI, confidence interval; Std, standard.

The meta‐analysis showed a positive effect on knee proprioception (SMD: 0.20; 95% CI: 0.10–0.30; *p* < 0.001) with no evidence of heterogeneity (*I* ^2^ = 0%, *p* = 0.48) (Figure 9). Although the pooled effect size was statistically significant, the clinical relevance of this finding remains uncertain because no validated MCID threshold has been established for proprioception outcomes following ACLR.

Therefore, while these findings suggest statistically detectable changes in knee laxity, functional performance and knee proprioception following AR, the clinical significance of these effects should be interpreted with caution. The observed improvements in several clinical outcomes remained below established MCID thresholds; however, the clinical meaningfulness of proprioceptive changes cannot be determined because a validated MCID threshold for proprioception following ACLR has not yet been established.

## DISCUSSION

The main findings of the present study were that statistically significant changes in knee anterior‐posterior laxity, proprioception and several functional performance outcomes, including IKDC, Lysholm, KOOS‐ADL and the single‐leg hop test, were observed following AR in individuals post‐ACLR. However, none of these changes reached clinically meaningful thresholds. AR did not consistently reduce knee pain or improve Tegner scores. Therefore, these findings suggest that although AR may lead to statistically detectable changes in selected objective outcomes, their clinical relevance appears limited.

The lack of clinically meaningful improvements in pain outcomes raises concerns regarding the effectiveness of AR in improving pain‐related recovery after ACL reconstruction. Clinical recommendations emphasize that post‐exercise pain greater than 2/10 on the numeric rating scale or any increase in joint effusion may indicate the need for load modification during rehabilitation [38, 40, 55]. Therefore, AR should be implemented cautiously in rehabilitation phases where pain remains a limiting factor [2, 61].

Although psychological and neuromuscular factors may contribute to rehabilitation outcomes following ACLR [43, 47], these factors were not directly evaluated in the included studies and should therefore be interpreted cautiously. Similarly, while AR is designed to improve explosive performance characteristics, such as rate of force development and reactive strength [51], the present findings do not allow conclusions regarding its effects on underlying pain mechanisms. Previous systematic reviews have reported that early or intensive plyometric interventions do not provide meaningful improvements in VAS or KOOS‐pain scores compared with conventional strengthening or closed kinetic chain programmes [8, 41].

Conversely, sensorimotor and proprioceptive training have shown potential benefits for pain reduction during early postoperative rehabilitation in some studies [36]. Given the moderate heterogeneity observed, the prediction interval may provide additional insight into the variability of treatment effects across studies beyond the *I* ^2^ statistic alone. Therefore, interpretations regarding the potential mechanisms underlying pain responses should remain tentative, as these mechanisms were not directly assessed in the included studies.

Moreover, the meta‐analysis suggested a reduction in anterior‐posterior knee laxity between the AR and standard rehabilitation groups. Although the pooled KT‐1000 result showed a statistically detectable difference, the magnitude of change was below the MCID, indicating limited clinical relevance. Improved Limb Symmetry Index (LSI) values were observed in individuals undergoing AR, which may reflect better functional symmetry rather than direct changes in joint stability [4, 27]. Ebert et al. reported that more than 90% of patients in the AR protocol achieved normal KT‐1000 values (<3 mm), while Christensen et al. found no differences between AR and standard rehabilitation groups in anterior‐posterior knee laxity [28].

Low heterogeneity suggests consistency across studies; however, differences in exercise protocols, participant characteristics and study designs may have influenced the results, warranting cautious interpretation. Therefore, these findings emphasize limited clinical relevance despite statistically detectable changes in knee laxity outcomes.

Additionally, AR resulted in statistically detectable changes but clinically trivial improvements in most functional outcomes in individuals post‐ACLR. These improvements may contribute to aspects of neuromuscular control, rate of force development and dynamic stability relevant to return‐to‐sport activities [14, 42]. These improvements may be related to functional adaptations associated with AR; however, specific neuromuscular mechanisms, including changes in rate of force development or stretch‐shortening cycle function, were not directly assessed in the included studies [19].

Moreover, heterogeneity was generally low; however, this may be influenced by the small number of included studies, which limits the robustness and interpretability of heterogeneity estimates. Moderate heterogeneity in KOOS symptoms and Tegner scores likely reflects differences in intervention protocols, follow‐up durations, participant activity levels or baseline characteristics. Variations in other functional outcomes may be attributed to differences in testing methods or intervention intensity. Although some outcomes reached statistically detectable change, most improvements fell below the established thresholds for MCID, indicating limited clinical relevance. These findings highlight the importance of considering MCID when interpreting results, ensuring a balanced perspective on the practical benefits of the interventions.

Additionally, the present meta‐analysis showed statistically detectable changes in knee proprioception; however, these changes did not reach clinically meaningful thresholds. These findings may reflect adaptations associated with high‐velocity rehabilitation exercises [22, 50]; however, the specific neuromuscular mechanisms underlying changes in proprioception were not directly evaluated in the included studies. Clinically, proprioceptive outcomes may require more specific perturbation‐based, multidirectional or sensorimotor training strategies that directly challenge joint position awareness and neuromuscular control [45].

No heterogeneity was observed across studies in proprioception outcomes. The clinical relevance of these proprioceptive changes remains uncertain because no validated MCID threshold has been established for knee proprioception following ACLR. Therefore, although AR demonstrated a statistically significant change in proprioception, the extent to which these changes translate into meaningful patient‐level benefits remains unclear. Overall, statistically detectable differences are unlikely to translate into meaningful functional or patient‐reported improvements.

The sensitivity analyses indicated that the robustness of the pooled findings varied across outcomes. Although AR demonstrated changes in several clinical outcomes, most of the pooled estimates were influenced by the exclusion of individual studies. In particular, the effects on knee laxity, IKDC score, KOOS‐ADL, Lysholm score, and knee proprioception were sensitive to the removal of specific studies, suggesting that these findings should be interpreted with caution. Additionally, the statistically significant effects observed for knee laxity and proprioception were no longer significant after removal of the De Carlo et al. [25] study. Given that this study contributed 1652 participants and represented a substantially larger sample size compared with the other included studies, the observed effects for these outcomes appear to be highly dependent on a single historical study. Furthermore, De Carlo et al. [25] was conducted during an earlier era of ACLR and rehabilitation, when surgical techniques, graft management, and rehabilitation approaches differed from contemporary clinical practice. Therefore, the current evidence supporting improvements in knee laxity and proprioception should be interpreted with considerable caution, and these findings require confirmation through modern, well‐designed RCTs. The influence of individual studies may be related to differences in study design, sample size, rehabilitation protocols, and outcome assessment methods. Moreover, several influential studies were non‐randomized designs, which may have contributed to variability in the pooled estimates. In contrast, the effect on the single‐leg hop test remained stable across sensitivity analyses, supporting greater confidence in this finding. Therefore, while AR may provide beneficial effects following ACLR, the magnitude and clinical relevance of improvements in some outcomes require further confirmation. Moreover, although sensitivity analyses were performed to evaluate the robustness of the findings, the pooled estimates should be interpreted cautiously because they represent a combination of randomized and non‐randomized evidence with different levels of inherent bias. Therefore, the certainty of the observed effects may be influenced by methodological differences between study designs.

This review has several important limitations that should be considered when interpreting the findings. First, despite our refined definition of AR, some heterogeneity remained across studies regarding rehabilitation content, progression criteria, loading strategies, supervision and rehabilitation milestones. Although all included interventions were initiated during the early postoperative phase following ACLR, variations in protocol design may have influenced the pooled estimates. Second, the included studies comprised both RCTs and non‐randomized studies. Although sensitivity analyses were performed to evaluate the robustness of the findings, subgroup analyses based on study design were not feasible due to the limited number of available studies. Therefore, the pooled estimates represent a combination of evidence generated from different methodological designs with varying risks of bias. This limitation may have affected the certainty of the conclusions, and future well‐designed RCTs are required to confirm the observed effects. Third, several outcomes were based on a limited number of studies, reducing statistical power and increasing uncertainty around pooled estimates. Fourth, one historical study [25] contributed a substantially larger sample size than the remaining studies (1652 participants) and had a major influence on the pooled estimates for knee laxity and proprioception. Sensitivity analyses demonstrated that removal of this study eliminated the statistical significance of these outcomes, indicating that the evidence supporting improvements in these domains remains uncertain and should be interpreted with considerable caution. Moreover, because this study was conducted in an earlier surgical and rehabilitation era, differences in ACLR techniques and rehabilitation protocols may limit the applicability of these findings to contemporary practice. Fifth, follow‐up duration varied considerably across studies, potentially affecting the comparability of outcome assessments and limiting conclusions regarding long‐term recovery. Sixth, adverse events, graft failure, reinjury, knee effusion and other rehabilitation‐related complications were inconsistently reported across the included studies. Therefore, the safety profile of AR and its potential effects on graft‐related outcomes could not be comprehensively evaluated and should be interpreted with a high degree of caution. Seventh, some heterogeneity existed regarding graft type. Different graft choices, including hamstring tendon and bone–patellar tendon–bone grafts, may influence postoperative recovery and functional outcomes. However, subgroup analyses based on graft type were not feasible because of incomplete reporting and the limited number of available studies. Finally, publication bias assessments should be interpreted cautiously because most meta‐analyses included fewer than ten studies, limiting the reliability and statistical power of Begg's and Egger's tests. Furthermore, although some statistically significant effects were observed, most patient‐reported and functional outcomes did not reach established MCID thresholds. In contrast, the clinical meaningfulness of proprioception changes remains uncertain due to the absence of a validated MCID threshold, and several findings were sensitive to individual‐study removal, suggesting that the clinical relevance of these outcomes should be interpreted cautiously.

Future high‐quality RCTs should adopt standardized definitions and protocols for AR, including clearly reported progression criteria, loading parameters, supervision strategies and rehabilitation milestones. Studies should incorporate larger sample sizes, longer follow‐up periods and consistent reporting of graft type, adverse events, graft failure, reinjury and other safety‐related outcomes. In addition, future research should combine patient‐reported outcomes with objective functional and biomechanical assessments to better determine whether statistically significant findings translate into clinically meaningful benefits for patients following ACLR.

## CONCLUSIONS

This systematic review and meta‐analysis suggest that AR may be associated with statistically detectable changes in knee laxity, functional outcomes and knee proprioception; however, these effects did not reach clinically meaningful thresholds and did not consistently extend to pain reduction. Therefore, the clinical value of AR in optimizing post‐ACLR recovery remains uncertain.

## AUTHOR CONTRIBUTIONS

**Rahman Sheikhhoseini**: Conceptualization; methodology; software; validation; investigation; data curation; writing the original draft; review and editing; supervision and project administration. **Mostafa Jalili Bafrouei**: Conceptualization; software; methodology; validation; formal analysis; investigation; data curation; writing the original draft; review and editing; supervision and project administration. **Hooman Minoonejad**: Conceptualization; validation; data curation; formal analysis and investigation. **Seyed Hamed Mousavi**: Conceptualization; validation; methodology; review and editing; data curation; formal analysis and investigation.

## CONFLICT OF INTEREST STATEMENT

The author declares no conflicts of interest.

## FUNDING INFORMATION

The authors have no funding to report.

## ETHICS STATEMENT

The authors have nothing to report.

## Supporting information

Supporting File 1.

Supporting File 2.

Supporting File 3.

Supporting File 4.

Supporting File 5.

Supporting File 6.

## Data Availability

All relevant data are included in the article.
