# Supplementary material for: Effects of accelerated rehabilitation after ACL reconstruction on clinical and functional outcomes: A systematic review and meta‐analysis evaluating clinical meaningfulness
Source: J Exp Orthop. 2026 Aug 6;13(3):e70878. doi: 10.1002/jeo2.70878 (PMC13446283; doi:10.1002/jeo2.70878)
Supplement: Supplementary file 1 [file JEO2-13-e70878-s001.docx]

Supplementary Material 5. Sensitivity analysis

Figure 2B*.* Sensitivity analysis comparing the accelerated rehabilitation group (left) and the standard rehabilitation group (right) in the knee pain.

Figure 3B*.* Sensitivity analysis comparing the accelerated rehabilitation group (left) and the standard rehabilitation group (right) in the KT-1000 score.

Figure 4B*.* Sensitivity analysis comparing the accelerated rehabilitation group (left) and the standard rehabilitation group (right) in the IKDC score.

Figure 5B*.* Sensitivity analysis comparing the accelerated rehabilitation group (left) and the standard rehabilitation group (right) in the KOOS-ADL score.

Figure 6B*.* Sensitivity analysis comparing the accelerated rehabilitation group (left) and the standard rehabilitation group (right) in the Lysholm score.

Figure 7B*.* Sensitivity analysis comparing the accelerated rehabilitation group (left) and the standard rehabilitation group (right) in the single-leg hop test.

Figure 8B*.* Sensitivity analysis comparing the accelerated rehabilitation group (left) and the standard rehabilitation group (right) in the Tegner score.

Figure 9B*.* Sensitivity analysis comparing the accelerated rehabilitation group (left) and the standard rehabilitation group (right) in the knee proprioception.
