# Supplementary material for: Effects of accelerated rehabilitation after ACL reconstruction on clinical and functional outcomes: A systematic review and meta‐analysis evaluating clinical meaningfulness
Source: J Exp Orthop. 2026 Aug 6;13(3):e70878. doi: 10.1002/jeo2.70878 (PMC13446283; doi:10.1002/jeo2.70878)
Supplement: Supplementary file 2 [file JEO2-13-e70878-s002.docx]

Supplementary Material 3. Risk of bias

Risk of bias for RCTs.

| **Study/question** | **Randomization process** | **Deviations from the intended interventions** | **Missing outcome data** | **Measurement of the outcome** | **Selection of the reported results** | **Overall** |  |  |
| --- | --- | --- | --- | --- | --- | --- | --- | --- |
| Beynnon et al. 2011 (13) | **+** | **?** | **+** | **+** | **+** | **?** |  |  |
| Cristiani et al. 2020 (59) | **+** | **?** | **+** | **+** | **+** | **?** |  |  |
| Christensen et al. 2013 (22) | **+** | **?** | **+** | **+** | **+** | **?** |  |  |
| Ebert et al. 2022 (28) | **+** | **?** | **+** | **+** | **+** | **?** |  |  |
| Elabd et al. 2024 (29) | **+** | **?** | **+** | **+** | **+** | **?** |  |  |
|  |  |  |  |  |  |  | **+** | Low risk |
|  |  |  |  |  |  |  | **-** | High risk |
|  |  |  |  |  |  |  | **?** | Some concerns |

Risk of bias for non-randomized studies.

| **Authors** | **Bias due to confounding** | **Bias in selection of participants into the study** | **Bias in classification of interventions** | **Bias due to deviations from intended interventions** | **Bias due to missing data** | **Bias in measurement of outcomes** | **Bias in selection of the reported result** | **Overall** |  |  |
| --- | --- | --- | --- | --- | --- | --- | --- | --- | --- | --- |
| **De carlo et al. 1992 (62)** | **+** | **+** | **?** | **+** | **?** | **+** | **+** | **?** |  |  |
| **Lee et al. 2016 (46)** | **+** | **+** | **+** | **+** | **?** | **+** | **+** | **?** |  |  |
| **Ok an et al. 2016 (6)** | **+** | **+** | **+** | **+** | **?** | **+** | **+** | **?** |  |  |
| **Gupta et al. 2017 (34)** | **+** | **+** | **+** | **+** | **?** | **+** | **+** | **?** |  |  |
| **Deichsel et al. 2023 (26)** | **+** | **+** | **?** | **+** | **?** | **+** | **+** | **?** |  |  |
|  |  |  |  |  |  |  |  |  | **+** | Low risk |
|  |  |  |  |  |  |  |  |  | **-** | High risk |
|  |  |  |  |  |  |  |  |  | **?** | Some concerns |
