# Supplementary material for: Effects of accelerated rehabilitation after ACL reconstruction on clinical and functional outcomes: A systematic review and meta‐analysis evaluating clinical meaningfulness
Source: J Exp Orthop. 2026 Aug 6;13(3):e70878. doi: 10.1002/jeo2.70878 (PMC13446283; doi:10.1002/jeo2.70878)
Supplement: Supplementary file 3 [file JEO2-13-e70878-s003.docx]

Supplementary Material 2. The results of quality assessment using the PEDro scale and the NOS

| Studies/PEDro Scale | Q2 | Q3 | Q4 | Q5 | Q6 | Q7 | Q8 | Q9 | Q10 | Q11 | Overall |
| --- | --- | --- | --- | --- | --- | --- | --- | --- | --- | --- | --- |
| Beynnon et al. 2011 (13) | 1 | 0 | 1 | 1 | 0 | 1 | 1 | 1 | 1 | 1 | 8/10 |
| Christensen et al. 2013 (22) | 1 | 0 | 1 | 0 | 0 | 0 | 0 | 1 | 1 | 1 | 5/10 |
| Cristiani et al. 2020 (59) | 1 | 1 | 1 | 1 | 0 | 0 | 1 | 1 | 1 | 1 | 8/10 |
| Ebert et al. 2022 (28) | 1 | 0 | 1 | 0 | 0 | 0 | 1 | 1 | 1 | 1 | 6/10 |
| Elabd et al. 2024 (29) | 1 | 1 | 1 | 1 | 0 | 0 | 1 | 1 | 1 | 1 | 8/10 |
| Studies/NOS* | Q1 | Q2 | Q3 | Q4 | Q5 | Q6 | Q7 | Q8 | - | - | - |
| Gupta et al. 2017 (34) | 1 | 0 | 1 | 2 | 0 | 1 | 1 | - | - | - | 6/9 |
| Ok an et al. 2016 (6) | 1 | 0 | 1 | 2 | 1 | 2 | 1 | - | - | - | 8/9 |
| Lee et al. 2016 (46) | 1 | 0 | 1 | 2 | 1 | 2 | 1 | - | - | - | 8/9 |
| De carlo et al. 1992 (62) | 1 | 1 | 1 | 1 | 0 | 1 | 0 | - | - | - | 5/9 |
| Deichsel et al. 2023 (26) | 1 | 1 | 1 | 1 | 0 | 1 | 0 | - | - | - | 5/9 |
