# Supplementary material for: Effects of accelerated rehabilitation after ACL reconstruction on clinical and functional outcomes: A systematic review and meta‐analysis evaluating clinical meaningfulness
Source: J Exp Orthop. 2026 Aug 6;13(3):e70878. doi: 10.1002/jeo2.70878 (PMC13446283; doi:10.1002/jeo2.70878)
Supplement: Supplementary file 5 [file JEO2-13-e70878-s005.docx]

Supplementary Material 1. Level of evidence

| Level of evidence | Description |
| --- | --- |
| Strong evidence | Pooled results from three or more studies, including a minimum of two high-quality studies, such as the PEDro scale for RCTs, the Newcastle-Ottawa scale (NOS) for non-RCTs, and which are statistically homogenous (p>0.05), or may be associated with statistically significant or non-significant pooled results. |
| Moderate evidence | Statistically significant pooled results from multiple studies, including at least one high-quality study, which are statistically heterogeneous (p<0.05); or from multiple low- or moderate-quality studies which are statistically homogenous (p>0.05); or statistically insignificant pooled results from multiple studies, including at least one high-quality study, which are statistically homogenous (p>0.05). An I² cut-off to aid interpretability. |
| Limited evidence | Results from multiple low- or moderate-quality studies, which are statistically heterogeneous (p<0.05), or from one high-quality study. An I² cut-off to aid interpretability. |
| Very limited evidence | Results from one low- or moderate-quality study. |
| Conflicting evidence | Pooled results that are insignificant and from multiple studies, regardless of quality, which are statistically heterogeneous (p<0.05, i.e., inconsistent). |

*I²* value of 25% or less as low; 50% as moderate; and 75% or more as high
