# Supplementary material for: Effects of accelerated rehabilitation after ACL reconstruction on clinical and functional outcomes: A systematic review and meta‐analysis evaluating clinical meaningfulness
Source: J Exp Orthop. 2026 Aug 6;13(3):e70878. doi: 10.1002/jeo2.70878 (PMC13446283; doi:10.1002/jeo2.70878)
Supplement: Supplementary file 6 [file JEO2-13-e70878-s006.docx]

sSupplementary Material 4. Detailed GRADE assessments for all outcomes are presented.

| **Certainty assessment** | | | | | | | **№ of patients** | | **Effect** | | **Certainty** |  |
| --- | --- | --- | --- | --- | --- | --- | --- | --- | --- | --- | --- | --- |
| **№ of studies** | **Study design** | **Risk of bias** | **Inconsistency** | **Indirectness** | **Imprecision** | **Other considerations** | **ACC exe** | **traditional** | **Relative (95% CI)** | **Absolute (95% CI)** |  |  |
| **IKDC (follow-up: range 12 weeks to 24 months)** | | | | | | | | | | | | |
| 5 | randomized studies and non-randomized | serious | not serious | not serious | not serious | strong association all plausible residual confounding would reduce the demonstrated effect dose response gradient | 109/214 (50.9%) | 0.0% |  | **0 fewer per 100** (from 0 fewer to 0 fewer) | ⨁⨁◯◯ Low |  |
| **Tegner score (follow-up: range 12 weeks to 24 months)** | | | | | | | | | | | | |
| 4 | randomized studies and non-randomized | serious | serious | not serious | serious | strong association all plausible residual confounding would reduce the demonstrated effect dose response gradient | 94/184 (51.1%) | 0.0% | **RR 0.68** (0.41 to 0.95) | **0 fewer per 1,000** (from 0 fewer to 0 fewer) | ⨁◯◯◯ Very low |  |
| **Lysholm score (follow-up: range 12 weeks to 24 months)** | | | | | | | | | | | | |
| 3 | randomized studies and non-randomized | serious | not serious | not serious | not serious | all plausible residual confounding would reduce the demonstrated effect dose response gradient | 63/124 (50.8%) | 0.0% | **RR 1.44** (0.08 to 2.81) | **0 fewer per 1,000** (from 0 fewer to 0 fewer) | ⨁⨁◯◯ Low |  |
| **KOOS activities daily living (follow-up: range 12 weeks to 24 months)** | | | | | | | | | | | | |
| 3 | randomized studies and non-randomized | serious | not serious | not serious | not serious | all plausible residual confounding would reduce the demonstrated effect dose response gradient | 89/176 (50.6%) | 0.0% | **RR 0.38** (0.01 to 0.75) | **0 fewer per 1,000** (from 0 fewer to 0 fewer) | ⨁⨁◯◯ Low |  |
| **Pain (follow-up: range 12 weeks to 24 months)** | | | | | | | | | | | | |
| 3 | randomized studies and non-randomized | serious | serious | not serious | serious | all plausible residual confounding would reduce the demonstrated effect dose response gradient | 101/199 (50.8%) | 0.0% | **RR -0.15** (-0.54 to 0.24) | **0 fewer per 1,000** (from 0 fewer to 0 fewer) | ⨁◯◯◯ Very low |  |
| **Single-leg hop test (follow-up: range 12 weeks to 24 months)** | | | | | | | | | | | | |
| 4 | randomized studies and non-randomized | serious | serious | not serious | not serious | strong association all plausible residual confounding would reduce the demonstrated effect dose response gradient | 159/314 (50.6%) | 0.0% | **RR 0.48** (-0.05 to 1.02) | **0 fewer per 1,000** (from 0 fewer to 0 fewer) | ⨁◯◯◯ Very low |  |
| **KT-1000 laxity (follow-up: range 12 weeks to 24 months)** | | | | | | | | | | | | |
| 3 | randomized studies and non-randomized | serious | not serious | not serious | not serious | strong association all plausible residual confounding would reduce the demonstrated effect dose response gradient | 1088 | 637 | - | **0**  (0 to 0 ) | ⨁⨁◯◯ Low |  |
| **Knee proprioception (follow-up: range 12 weeks to 24 months)** | | | | | | | | | | | | |
| 4 | randomized studies and non-randomized | serious | not serious | not serious | not serious | all plausible residual confounding would reduce the demonstrated effect dose response gradient | 95 | 94 | - | **0**  (0 to 0 ) | ⨁⨁◯◯ Low |  |
